# Host Cytokine Responses of Pigeons Infected with Highly Pathogenic Thai Avian Influenza Viruses of Subtype H5N1 Isolated from Wild Birds

**DOI:** 10.1371/journal.pone.0023103

**Published:** 2011-08-03

**Authors:** Tsuyoshi Hayashi, Yasuaki Hiromoto, Kridsada Chaichoune, Tuangthong Patchimasiri, Warunya Chakritbudsabong, Natanan Prayoonwong, Natnapat Chaisilp, Witthawat Wiriyarat, Sujira Parchariyanon, Parntep Ratanakorn, Yuko Uchida, Takehiko Saito

**Affiliations:** 1 Thailand-Japan Zoonotic Diseases Collaborating Center (ZDCC), Kasetklang, Chatuchak, Bangkok, Thailand; 2 Research Team for Zoonotic Diseases, National Institute of Animal Health, National Agriculture and Food Research Organization (NARO), Kannondai, Tsukuba, Ibaraki, Japan; 3 The Monitoring and Surveillance Center for Zoonotic Diseases in Wildlife and Exotic Animals, Faculty of Veterinary Science, Mahidol University, Salaya, Phuttamonthon, Nakhon Pathom, Thailand; 4 National Institute of Animal Health, Kasetklang, Chatuchak, Bangkok, Thailand; Indian Institute of Science, India

## Abstract

Highly pathogenic avian influenza virus (HPAIV) of the H5N1 subtype has been reported to infect pigeons asymptomatically or induce mild symptoms. However, host immune responses of pigeons inoculated with HPAIVs have not been well documented. To assess host responses of pigeons against HPAIV infection, we compared lethality, viral distribution and mRNA expression of immune related genes of pigeons infected with two HPAIVs (A/Pigeon/Thailand/VSMU-7-NPT/2004; Pigeon04 and A/Tree sparrow/Ratchaburi/VSMU-16-RBR/2005; T.sparrow05) isolated from wild birds in Thailand. The survival experiment showed that 25% of pigeons died within 2 weeks after the inoculation of two HPAIVs or medium only, suggesting that these viruses did not cause lethal infection in pigeons. Pigeon04 replicated in the lungs more efficiently than T.sparrow05 and spread to multiple extrapulmonary organs such as the brain, spleen, liver, kidney and rectum on days 2, 5 and 9 post infection. No severe lesion was observed in the lungs infected with Pigeon04 as well as T.sparrow05 throughout the collection periods. Encephalitis was occasionally observed in Pigeon04- or T.sparrow05-infected brain, the severity, however was mostly mild. To analyze the expression of immune-related genes in the infected pigeons, we established a quantitative real-time PCR analysis for 14 genes of pigeons. On day 2 post infection, Pigeon04 induced mRNA expression of Mx1, PKR and OAS to a greater extent than T.sparrow05 in the lungs, however their expressions were not up-regulated concomitantly on day 5 post infection when the peak viral replication was observed. Expressions of TLR3, IFNα, IL6, IL8 and CCL5 in the lungs following infection with the two HPAIVs were low. In sum, Pigeon04 exhibited efficient replication in the lungs compared to T.sparrow05, but did not induce excessive host cytokine expressions. Our study has provided the first insight into host immune responses of pigeons against HPAIV infection.

## Introduction

The highly pathogenic avian influenza virus (HPAIV) of subtype H5N1, that is currently spread worldwide was first isolated from domestic goose in Guangdong Province, China in 1996 [1]. The following year, sporadic outbreaks of H5N1 HPAIVs occurred in poultries across Hong Kong and were accompanied by human infections that resulted in the deaths of 6 of 18 Hong Kong residents infected with the virus [2]. HPAIVs were thought to cause lethal infections only in gallinaceous birds such as chickens, but not in domestic and wild waterfowls. In late 2002, however, H5N1 HPAIV outbreaks in Hong Kong occurred in waterfowls and wild birds, resulting in the deaths of many resident avian species including ducks, geese, swans, pigeons and tree sparrows [3]. These H5N1 HPAIVs, however, were reported to differ antigenetically from those isolated between 1997 and 2001, and were lethal to ducks in a laboratory experiment [4]. In 2005, H5N1 HPAI outbreaks in wild migratory waterfowls that occurred at Qinghai Lake, China, led to the die-off of migratory birds [5]. In Thailand, HPAIVs were isolated from mammalian species including tigers, leopards, dogs and cats and wild birds including open-bill storks, pigeons and tree sparrows during the HPAI outbreaks in poultries in 2004–2005 [6]–[11]. The Asian H5N1 HPAIVs isolated from humans in 2004 have been reported to have increased pathogenicity in ferrets compared to HPAIVs isolated in 1997 [12]. These reports suggest that current HPAIVs appear to be more lethal to mammals and wild birds compared to those isolated before 2001.

There are several reports on the virulent mechanisms of HPAIVs in birds, particularly chickens. Virulent H5N1 HPAIV, but not avirulent virus, was reported to inhibit mRNA expression of IFNα/β in chick embryonated cells. This indicated that the pathogenicity of HPAIVs in chickens is influenced by their ability to antagonize host IFN responses [13], [14]. Wasilenko et al demonstrated that earlier death of chickens infected with H5N1 HPAIVs was associated with efficient viral replication in the lungs and spleens accompanied by up-regulation of antiviral cytokines such as IFNα, IFNγ and orthomyxovirus resistance gene 1 (Mx1) [15]. Suzuki et al compared host cytokine responses towards two H5N1 HPAIVs with different mean death times (MDT) in virus infected chickens [16]. They suggested that efficient viral replication accompanied by destruction of innate immune responses in chickens contributed to increased pathogenecity of the HPAIVs. Barber et al reported the relationship between influenza virus sensor RIG-I and virulence of H5N1 HPAIV in ducks, which are believed to be more resistant to HPAIV infection than chickens [17]. They demonstrated that RIG-I is present in ducks, but appears to be absent in chickens. Also, a H5N1 HPAIV potently induced mRNA expression of RIG-I in the lungs of an infected duck. Interestingly, chicken fibroblast cell line DF-1 expressing duck RIG-I inhibited HPAIV replication, suggesting that duck RIG-I has a mechanism for inducing resistance against HPAIV infections [17]. Apoptosis is also considered a factor contributing to the pathogenicity of HPAIV in avian species. In fact, Ito et al showed that virulent avian influenza A virus induced apoptosis in the liver, kidney and brain of infected chickens [18]. Ueda et al reported that H5N1 HPAIV induced apoptosis to a larger extent than the low pathogenic avian influenza virus (LPAIV) in duck embryonic fibroblasts (DEF) [19].

Pigeons are often sold in live-bird markets across Asia, and are also commonly found in outdoor poultries [20]. Previous studies have showed total resistance in pigeons to the Hong Kong H5N1 HPAIV that was isolated in 1997, and concluded that pigeons played no role in the transmission of HPAIV to other species [21], [22]. Recent studies, however, demonstrated that this may no longer be the case. Several Asian H5N1 HPAIVs isolated during 2002–2005 were shown to cause infection in pigeons and some cases were lethal. This difference is likely related to the fact that recent HPAIVs have been found to have increased pathogencity towards wild birds compared to those isolated prior to 2001 as mentioned above [23]–[25]. Biswas et al reported that close contact with pigeons was one of the risk factors for HPAIV infection of backyard chickens in Bangladesh [26]. Pigeons inside a victim's home were suspected to be the source of infection in one fatal case of HPAIV infection in Indonesia (http://www.who.int/csr/don/2006_08_09/en/index.html). In another fatal case in Indonesia, the victim had cleaned pigeon droppings from blocked roof gutters of his home shortly before onset of the illness (http://www.who.int/csr/don/2006_05_29/en/). Together, it suggested that HPAIV infection of pigeons could be a potential threat in both veterinary and human public healths.

Pigeons posses abundant sialic acid linked to galactose by an α2,6 linkage (SAα2,6Gal), a well-recognized receptor for the human influenza virus in the respiratory tract including the pharynx, trachea, bronchioles and alveoli. On the other hand, sialic acid linked to galactose by an α2,3 linkage (SAα2,3Gal) is predominant in chickens [27]. The suggestion that pigeons are less susceptible to HPAIV infection was based on the fact that pigeons have few SAα2,3Gal receptors in the respiratory tract [27]. Detailed analysis of host immune responses of pigeons against viral infection has not been performed due to difficulties attributed mainly to the fact that only few host immune-related genes have been characterized in pigeons and the sequences have not been disclosed [28], [29].

In this study, in order to understand host immune responses against HPAIV infection in pigeons, we inoculated pigeons with two H5N1 HPAIVs isolated from wild birds during the HPAI outbreaks in poultries in 2004–2005. Then, we compared lethality, viral distribution and pathology in the infected pigeons. Next, we determined partial sequences of 14 host immune-related genes to set up quantitative reverse transcriptase real-time PCR on those genes. Expression of the host immune-related genes in lungs and brains of pigeons inoculated with two HPAIVs were then examined by the established PCR.

## Results

### Lethality, viral shedding and distribution in pigeons infected with two HPAIVs

**Figure 1:**
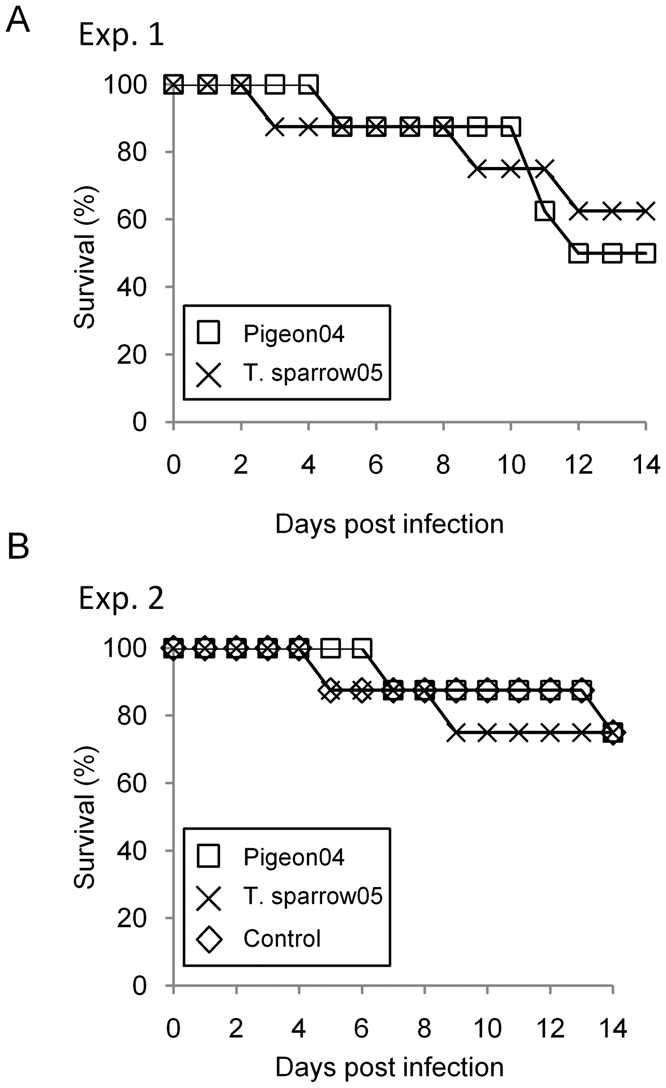
Lethality of two Thai HPAIVs in pigeons. Groups of eight adult rock pigeons were inoculated intranasally with 10^6^ EID_50_ of either of the two HPAIVs or mock infected with medium. After inoculation, the pigeons were monitored daily for clinical signs up to day 14 post infection.

Two groups of pigeons were inoculated intranasally with Pigeon04 or T.sparrow05 in the first experiments shown in Fig. 1A. Four of 8 pigeons in the Pigeon04-infected group, whereas 3 in the T.sparrow05-infected group died by day 14 post infection (Fig. 1A). The virus was recovered from the lungs, brain and tracheal swab in 3 of 4 dead pigeons in the Pigeon04-infected group, and the mean viral titers were 10^5.5^EID_50_/g, 10^4.7^EID_50_/g and 10^4.1^EID_50_/mL, respectively. Only 1 of the dead pigeons shed the virus in the cloacal swab with a titer of 10^4.1^EID_50_/mL. The virus was not recovered from either lungs, brain, tracheal nor cloacal swab from 1 of the dead pigeons in the Pigeon04-infected group and 2 in the T.sparrow05-infected group. Two of 4 pigeons in the Pigeon04-infected group that survived were seroconverted to H5 HA antigen on day 14 post infection, and the mean titers were 28 (Table 1). In contrast, no H5 HA-virus specific antibody was detected in the sera collected from 5 pigeons in the T.sparrow05-infected group that survived (Table 1). From the above experiment it was difficult to determine whether viral infection was the cause of death, therefore, we carried out an additional survival experiment using uninfected pigeons as the control (Fig. 1B). Two pigeons in each group died by day 14 post infection. However, the viruses were not recovered from any organ as well as the tracheal and cloacal swabs from the control pigeons. None of the control pigeons that survived was seroconvereted to the virus on day 14 post infection, suggesting that environmental stress elements in the experiment may have caused the death of the pigeons. In the virus-infected groups, 4 of 6 surviving pigeons were seroconverted to H5 HA antigen, and the mean titers in Pigeon04- and T.sparrow05-infected group were 24 and 57, respectively (Table 1). Both viruses were recovered from the lungs, brains and rectums in 1 to 2 dead pigeons (data not shown). Pigeon04 and T.sparrow05 were recovered from the tracheal swab in 1 to 2 dead pigeons and the titer was 10^1.2^EID_50_/mL and 10^4.2^EID_50_/mL, respectively. In the cloacal swab, only Pigeon04 was recovered with the titer of 10^2.9^EID_50_/mL. None of 6 pigeons that survived in the Pigeon04- or T.sparrow05-infected group shed the virus in the tracheal and cloacal swabs on day 14 post infection. These data showed that though the two Thai HPAIVs used in this study did not appear to produce a lethal outcome.

**Table 1:** HI assay of sera collected from the surviving pigeons.

Group	Seroconversion^a^	HI titers^b^				
		#1	#2	#3	#4	Average
Pigeon04, Exp1	2/4^c^	40	20			28
Pigeon04, Exp2	4/6	40	10	40	20	24
T.sparrow05, Exp1	0/5					
T.sparrow05, Exp2	4/6	80	20	80	80	57
Control, Exp2	0/6					

a Results are obtained from sera collected on day 14 post infection.

b Detection limit of the HI titers is 10.

c No. of seroconverted birds/total are recorded.

Next, we measured viral titers in the trachea and cloacal swabs collected from 3 pigeons sacrificed on days 2, 5 and 9 post infection. In the tracheal swabs, Pigeon04 were shed in all 3 pigeons on day 5 post infection, and mean viral titers were 10^2.8^EID_50_/mL (Table 2). In contrast, only 1 of 3 pigeons shed T.sparrow05 with the titer of 10^2.7^EID_50_/mL on day 5 post infection (Table 2). On day 9 post infection, Pigeon04 and T.sparrow05 were isolated from 1 pigeon, and the titers were 10^3.4^EID_50_/mL and 10^3.5^EID_50_/mL, respectively (Table 2). In the cloacal swabs, Pigeon04 was isolated from 1 of 3 pigeons on days 2, 5 and 9 post infection, and the titers were 10^3.7^EID_50_/mL, 10^2.4^EID_50_/mL and 10^3.4^EID_50_/mL, respectively (Table 2). None of the pigeons shed T.sparrow05 in the cloacal swab on the collection days (Table 2).

**Table 2:** Viral shedding and replication in pigeons infected with the Thai HPAI viruses.

Virus	Days p.i.	Mean viral titer (log_10_ EID_50_/g±SD) in^a^						Mean viral titer (log_10_ EID_50_/mL±SD) in^a^	
		Brain	Lung	Spleen	Liver	Kidney	Rectum	Tracheal	Cloacal
Pigeon04	2	1/3^b^ (3.0)^c^	3/3 (3.4±0.8)	2/3 (2.7, 3.4)	<	2/3 (3.5, 5.0)	2/3 (4.7, 3.7)	<	1/3 (3.7)
	5	2/3 (5.2, 6.2)	3/3 (5.3±1.2)	2/3 (5.0, 4.2)	3/3 (3.8±0.9)	2/3 (4.4, 5.0)	3/3 (4.7±0.2)	3/3 (2.8±0.8)	1/3 (2.4)
	9	1/3 (7.4)	1/3 (7.0)	1/3 (5.9)	<	1/3 (6.7)	1/3 (4.2)	1/3 (3.4)	1/3 (3.4)
T.sparrow05	2	<^d^	<	<	<	<	<	<	<
	5	2/3 (4.7, 5.2)	2/3 (4.7, 3.5)	1/3 (3.7)	1/3 (3.7)	2/3 (4.7, 3.2)	2/3 (4.4, 3.2)	1/3 (2.7)	<
	9	<	1/3 (4.3)	1/3 (3.7)	<	1/3 (5.4)	1/3 (3.0)	1/3 (3.5)	<

a Three pigeons from each group were killed on days 2, 5, 9 post infection, and viral titers in the indicated organs and swabs were determined. The detection limit of titers in organs and swabs were 10^2.2^ EID_50_/g and 10^0.7^ EID_50_/mL, respectively.

b No. of virus-recovered birds/total are recorded.

c When virus is not recovered from all 3 birds, individual titers are recored.

d Virus is not recovered from all 3 samples.

We also determined viral titers in the internal organs such as the lungs, spleen, brain, liver, kidney and rectum collected from the 3 pigeons sacrificed on days 2, 5 and 9 post infection. On day 2 post infection, Pigeon04 was recovered from the lungs of all 3 pigeons with a mean virus titer of 10^3.4^EID_50_/g (Table 2). This virus was also recovered in multiple organs including the brain, spleen, kidney and rectum in 1 or 2 pigeons with titers ranging from 10^3.0^∼10^5.0^EID_50_/g (Table 2). In contrast, T.sparrow05 was not recovered from such organs (Table 2). On day 5 post infection, Pigeon04 replicated in the lungs more efficiently than on day 2 post infection and the mean viral titer was 10^5.3^EID_50_/g (Table 2). This virus effectively spread to all extrapulmonary organs examined with titers ranging from 10^3.8^∼10^5.7^EID_50_/g in 2 to 3 pigeons (Table 2). T.sparrow05 replicated in the lungs to a less extent than Pigeon04 in 2 pigeons and mean virus titer was 10^4.1^EID_50_/g (Table 2). This virus spread to multiple extrapulmonary organs such as the brain, spleen, liver, kidney and rectum with titers, ranging from 10^3.7^∼10^5.0^EID_50_/g in 1 to 2 pigeons even though its efficiency was at lower levels compared to Pigeon04 (Table 2). On day 9 post infection, Pigeon04 was recovered from the lungs, brain, spleen, kidney and rectum with titers ranging 10^4.2^∼10^7.4^EID_50_/g in 1 pigeon (Table 2). T.sparrow05 was also recovered from the lungs, spleen, kidney and rectum with titers ranging 10^3.0^∼10^5.4^EID_50_/g in 1 pigeon (Table 2). Taken together, both viruses could cause systemic infection in pigeons and spread to multiple extrapulmonary organs but more efficiently for Pigeon04 than T.sparrow05.

### Host cytokine responses in the lungs and brain of pigeons infected with two Thai HPAIVs

In order to examine host cytokine responses of pigeons infected with the HPAIVs, we needed to determine the partial sequences of the immune-related genes in pigeons to set up quantitative reverse transcriptase real-time PCR analysis. The sequence for the 14 genes we chose for the examination (Table 3) was not available in public databases. The sequences were determined by RT-PCR of RNA from pigeons with primer sets designed based upon a conserved sequence of regions for orthologs in other species that was available in the database as described in the Materials and Methods. Sequences determined in this study revealed more than 70% of identities to those of other birds, such as chicken, zebra finch and duck (Table 3). Interestingly, the similarities of genes identified between pigeon and other birds varied individually, that is, TLR3, TGFβ3, SMAD7, Caspase3 and ApaF had more than 90% between them, whereas IFNα and PKR had only 70∼80% similarities (Table 3).

**Table 3:** Summary of immune-related genes of pigeons determined in this study.

Gene	Product size, bp	homology (%)	Accesion No.
RIG-I	804	Zebra Finch (87%)	AB618532
		Duck (84%)	
TLR3	709	Zebra Finch (93%)	AB618533
		Chicken (90%)	
IFNα	304	Zebra Finch (77%)	AB618534
		Duck (69%)	
		Chicken (67%)	
Mx1	354	Duck (86%)	AB618535
		Chicken (82%)	
PKR	762	Zebra Finch (79%)	AB618536
		Chicken (72%)	
OAS	136	Chicken (84%)	AB618537
IL6	360	Zebra Finch (87%)	AB618538
		Duck (87%)	
		Chicken (81%)	
CCL5	123	Zebra Finch (89%)	AB618539
		Chicken (88%)	
IL10	228	Zebra Finch (86%)	AB618540
		Chicken (83%)	
		Turkey (82%)	
TGFβ3	271	Zebra Finch (95%)	AB618541
		Chicken (93%)	
SMAD7	200	Zebra Finch (98%)	AB618542
		Chicken (93%)	
Caspase3	335	Zebra Finch (94%)	AB618543
		Chicken (90%)	
ApaF	1159	Zebra Finch (92%)	AB618544
		Chicken (91%)	
Bcl2	508	Chicken (84%)	AB618545
β-actin	1049	Chicken (97%)	AB618546
		Duck (97%)	
		Turkey (95%)	

**Figure 2:**
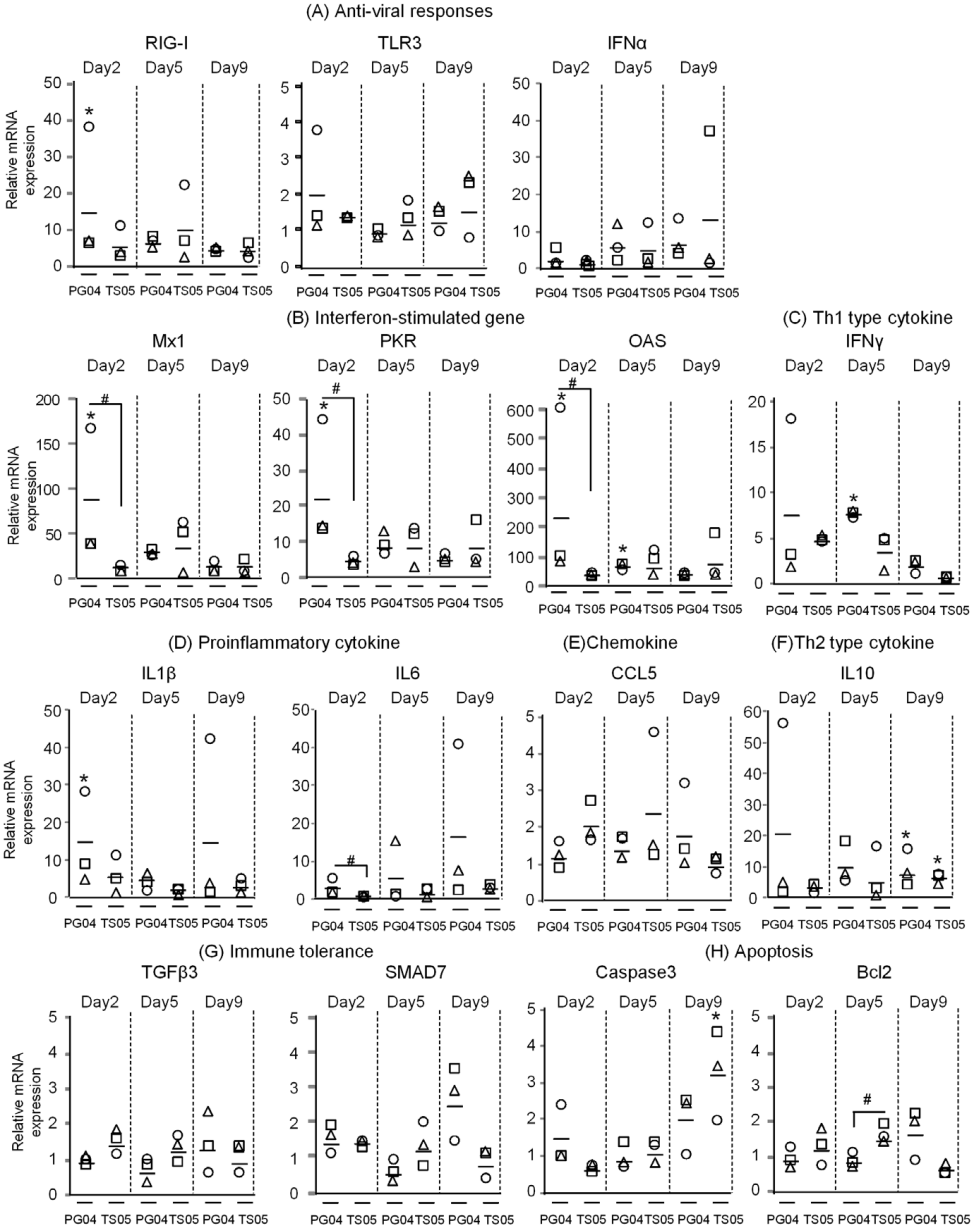
Host gene responses in the lungs of the infected pigeons against the two HPAIVs. Groups of nine pigeons were inoculated with 10^6^ EID_50_ of either of the two HPAIVs or mock infected with medium. On days 2, 5, 9 post infection, three pigeons in each group were euthanized, dissected and RNA was extracted as mentioned in Materials and Methods. The mRNA levels are presented as the mean values ± standard deviation. Statistical analysis was performed for the Pigeon04-infected, T.sparrow05-infected and uninfected groups by ANOVA followed by Turkey analysis. The asterisks indicate that the virus-infected group was significantly different from the uninfected group (p<0.05). Sharps indicate that the Pigeon04-infected group was significantly different from the T.sparrow05-infected group (p<0.05). PG04, Pigeon04: TS05, T.sparrow05.

Next, mRNA expressions of cytokines and apoptosis-related genes in the lungs and brains against viral infection were quantified by real-time PCR analysis with the primer pairs designed in this study (Table 4). We measured mRNA expression levels of 7 groups categorized as follows: anti-viral response (RIG-I, TLR3 and IFNα), interferon-stimulated gene (ISGs: Mx1, PKR and OAS), Th1 type cytokine (IFNγ), pro-inflammatory cytokine (IL1β and IL6), chemokine (IL8 and CCL5), Th2 type cytokine (IL10), immune tolerance (TGFβ3 and SMAD7), and apoptosis (Caspase 3, ApaF and Bcl2). On day 2 post infection, Pigeon04 induced mRNA expressions of Mx1, PKR and OAS in the lungs of the infected pigeons to a greater extent than T.sparrow05 (Fig. 2). The expressions of RIG-I, TLR3, IFNγ, IL1β, IL6 and IL10 were induced by Pigeon04 in 1 lung sample in which the expressions of Mx1, PKR and OAS were highly up-regulated (Fig. 2). On day 5 post infection, Pigeon04 induced the expression of IFNγ to a greater extent than T.sparrow05 (Fig. 2). In contrast, cytokine expressions in the lungs of the T.sparrow05-infected pigeons were at relatively low levels compared to those of the Pigeon04-infected pigeons on days 2 and 5 post infection (Fig. 2). On day 9 post infection, Pigeon04 induced mRNA expressions of IL1β and IL6 in 1 lung sample (Fig. 2). On the other hand, T.sparrow05 significantly induced the expression of Caspase 3 compared to the control group (Fig. 2). IFNα was also up-regulated in 1 lung sample in which the expression of Caspase 3 was up-regulated (Fig. 2). The expressions of TGFβ, SMAD7, IL8 and ApaF in the lungs of the Pigeon04- or T.sparrow05- infected groups remained at low levels throughout the collection periods (Fig. 2 and data not shown).

**Table 4:** Primer sequences used in the Quantitative real-time PCR analysis.

Gene	Sequence (5′→3′)	Product size, bp
RIG-I		92
Forward primer	TGAACTTGCACAGCCTGCTA	
Reverse primer	CACAAATCAGAATCGCCACA	
TLR3		80
Forward primer	CCCAAGCCTTAGAAAACTGATG	
Reverse primer	GCAGAGGATGAAAAGGTGAAG	
IFNα		96
Forward primer	CCAGCACCTCTTGCAAATCC	
Reverse primer	CTGTGGTGGTGGAGGCTGTT	
Mx1		86
Forward primer	TTACCAGGACATCAGCAGAGA	
Reverse primer	TAGTACCAGCCACGACATCC	
PKR		108
Forward primer	CCTGTTGGTGAAGGTGGTT	
Reverse primer	TTCACGCCTCACATCTCTTG	
OAS		81
Forward primer	TCCCAGCTTCACAGAACTGC	
Reverse primer	TACTTGACGAGGCGCAGGAG	
IFNγ		136
Forward primer	CAAGTCAAAGGCGCACGTC	
Reverse primer	GCGTTGAGTTTTCAAGTCATTC	
IL1β		136
Forward primer	GAGGAAGCCGACATCAGGAG	
Reverse primer	GGGACGTGCAGATGAACCAG	
IL6		107
Forward primer	AGCGTCGATTTGCTGTGCT	
Reverse primer	GATTCCTGGGTAGCTGGGTCT	
IL8		74
Forward primer	CCACTGCTCCCTGGGTCCAG	
Reverse primer	CACAGTGGTGCATCAGAATTGA	
CCL5		95
Forward primer	GTGAAGGACTATTTCTACACCAGCA	
Reverse primer	GCGTCAGGGTTTGCACAGA	
IL10		93
Forward primer	TGATGAACTTAGCATCCAGCTACTC	
Reverse primer	AACTGCATCATCTCCGACACA	
TGFβ3		106
Forward primer	AGGACCTTGGCTGGAAATG	
Reverse primer	ACCGTGCTGTGAGTGGTGT	
SMAD7		80
Forward primer	CTCGGACAACAAAAGCCAAC	
Reverse primer	CGTCCACTTCCTTGGTGAG	
Caspase3		82
Forward primer	GATGGCCCTCTTGAACTGAA	
Reverse primer	AGAGCTTGGGTTTTCCTGCT	
ApaF		104
Forward primer	TTAAATGGGCACCTTCTTGG	
Reverse primer	GCACGTAACTTGGCTTGTTG	
Bcl2		102
Forward primer	CGAGTTTGGTGGTGTGATGT	
Reverse primer	AGGTGCCGGTTCAGGTACT	
β-actin		95
Forward primer	AGGCTACAGCTTCACCACCAC	
Reverse primer	CCATCTCCTGCTCAAAATCCA	

**Figure 3:**
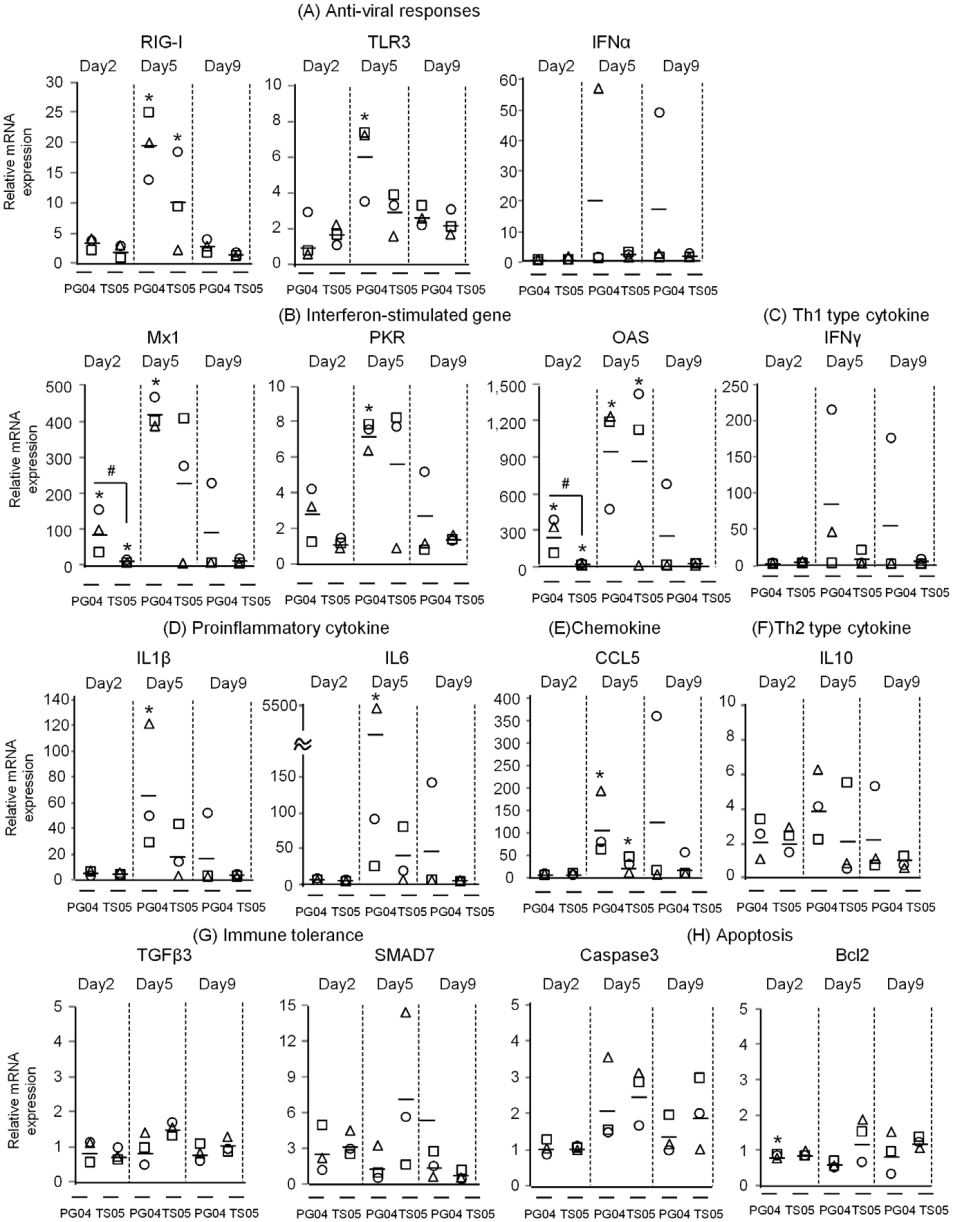
Host gene responses in the brains of the infected pigeons against the two HPAIVs. The mRNA levels are presented as the mean values ± standard deviation. The asterisks indicate that the virus-infected group was significantly different from the uninfected group (p<0.05). Sharps indicate that the Pigeon04-infected group was significantly different from the T.sparrow05-infected group (p<0.05). PG04, Pigeon04: TS05, T.sparrow05.

On day 2 post infection, Pigeon04 induced the expressions of Mx1 and OAS in the brain of the infected pigeons to a greater extent than T.sparrow05 and the situation was the same for the lungs (Figs. 2 and 3). On day 5 post infection, both viruses induced the expressions of RIG-I, Mx1 and OAS (Fig. 3). Pigeon04 induced the expressions of IL1β, IL6 and CCL5 to a greater extent than T.sparrow05 (Fig. 3). Messenger RNAs of IFNα was also up-regulated in 1 brain sample in which mRNAs of IL1β and IL6 were markedly increased (Fig. 3). On day 9 post infection, Pigeon04 markedly induced mRNA expressions of several cytokines such as IFNα, IFNγ, IL1β, IL6, IL10 and CCL5 in 1 brain sample, whereas T.sparrow05 did not induce the expression of any cytokine (Fig. 3). These data showed that Pigeon04 induced the mRNA expressions of interferon-stimulated genes such as Mx1 and OAS to a greater extent compared to T.sparrow05 in the lungs and brain on day 2 post infection. However, the mRNA expression of each gene in the lungs and brain following infection with the two HPAIVs appeared to vary.

### Correlation between viral replication or clinical sign and gene expression in the lungs and brain of pigeons infected with two HPAIVs

**Figure 4:**
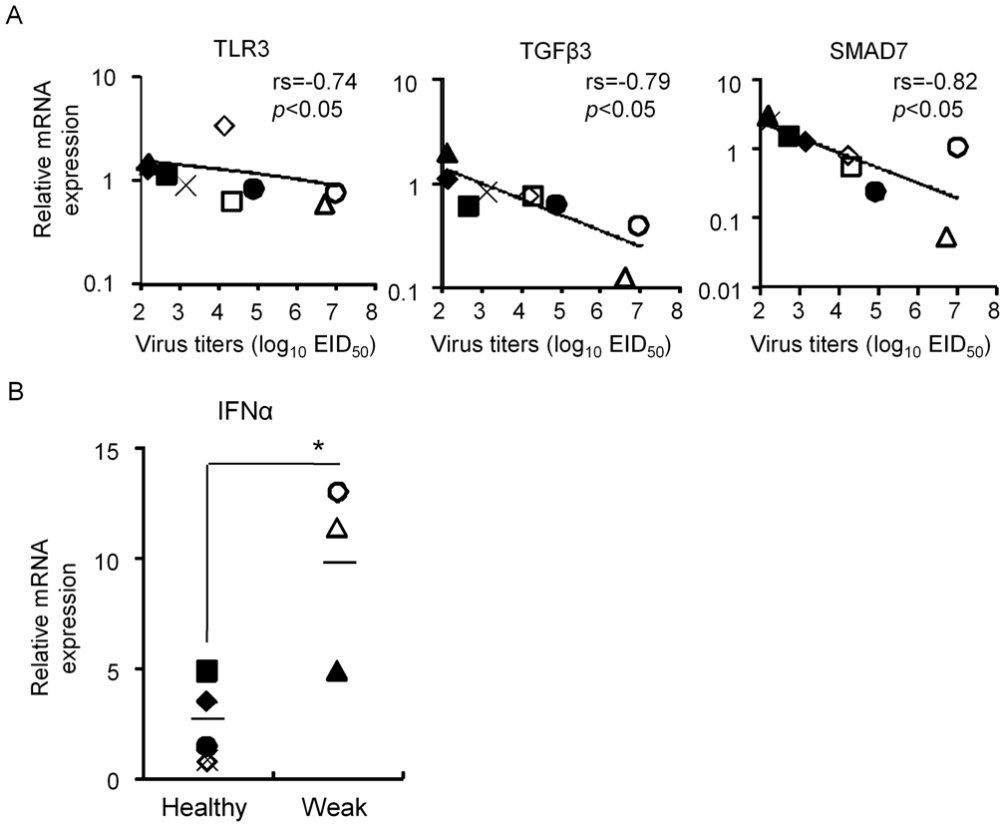
Correlation between host gene expression in the lungs of the infected pigeons and viral replication or clinical signs against Pigeon04 infection. (A) Correlation between host gene expression and viral replication in the lungs of nine individual pigeons collected on days 2, 5 and 9 post infection after Pigeon04 inoculation. Statistical analysis was performed by Spearman's rank correlation coefficient test. Correlation coefficients (rs) and P values are given in each graph. (B) Correlation between host gene expression and clinical sign of the infected pigeons. Asterisks indicate a statistically significant difference (*p<0.05 by Student's *t* test).

**Figure 5:**
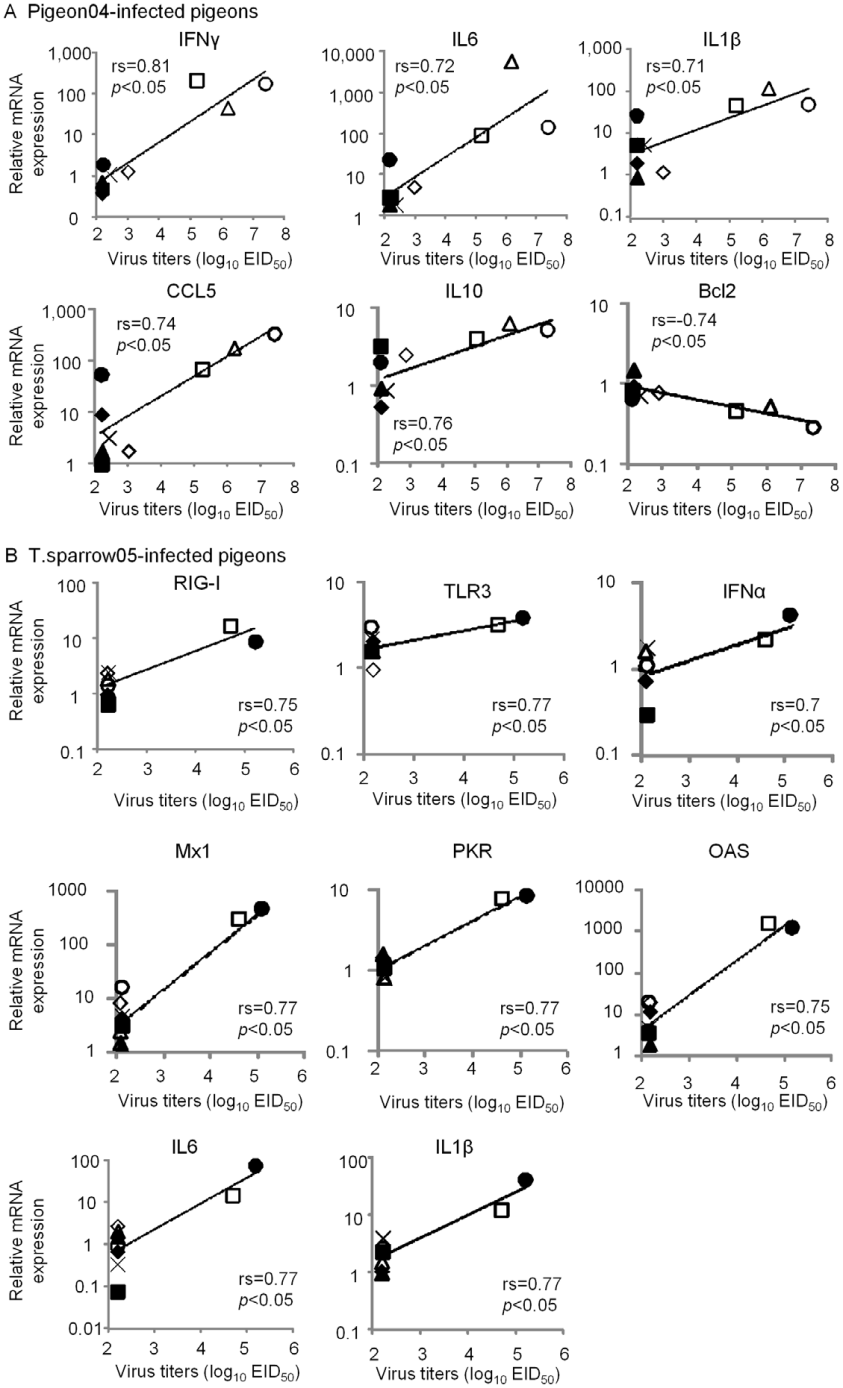
Correlation between host gene expression and viral replication in the brains of pigeons infected with Pigeon04 (A) and T.sparrow05 (B). Correlation coefficients (rs) and P values are given in each graph.

Our results demonstrated that the induction of host immune-related genes was accompanied by replication of the HPAIVs in the lungs and brain but their responses likely varied in each infected pigeon (Figs. 2 and 3). Therefore, to clarify the relationship between viral replication and host cytokine response against infection of the HPAIVs at an individual level, we assessed the correlation between virus replication and gene expression of the lungs and brain collected from 9 individual pigeons on days 2, 5 and 9 post infection in the Pigeon04- and T.sparrow05-infected groups. The mRNA expressions of TLR3, TGFβ3 and SMAD7 in the lungs following infection with Pigeon04 were negatively correlated with viral replication (Fig. 4A). We also tested the association between clinical signs of the infected pigeons and gene expressions. One pigeon in the T.sparrow05-infected group developed clinical symptoms including decreased activity and neurological signs such as torticollis on days 5 and 9 post infection (data not shown). One and 2 pigeons in the Pigeon04-infected group also developed symptoms on days 5 and 9 post infection, respectively (data not shown). In particular, 1 pigeon infected on day 9 post infection developed neurological disorders (data not shown). As shown in Fig. 4B, Pigeon04 significantly induced IFNα in the pigeons that developed clinical signs compared to those that did not. In the brain, mRNA expressions of IL6 and IL1β in the brain infected with two HPAIVs were positively correlated with viral replications (Fig. 5). Pigeon04 induced the expressions of IFNγ, CCL5 and IL10 in a viral replication-dependent manner, whereas T.sparrow05 induced the expressions of RIG-I, TLR3, IFNα, Mx1, PKR and OAS in a viral titer-dependent manner (Fig. 5). No significant association between clinical sign of the infected pigeons and the corresponding gene expression was found (data not shown). These data suggested that both HPAIVs likely induced the expression of different sets of host-immune related genes dependent on viral replication in the brain of the infected pigeons, along with the up-regulation of pro-inflammatory cytokines in a viral replication-dependent manner in the brains of the infected pigeons.

### Histopathological analysis in the lungs and brains of pigeons infected with two Thai HPAIVs

**Figure 6:**
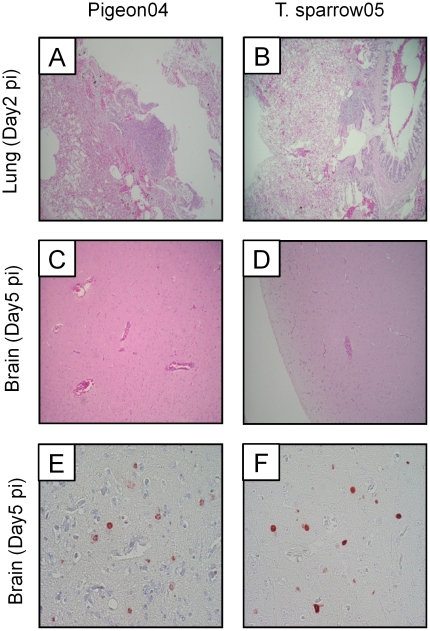
Histological findings of pigeons infected with two HPAIVs. (A–D) Representative hematoxylin- and eosin-stained images in the lungs and brains infected with Pigeon04 (A and C) and T.sparrow05 (B and D). Lung sections of pigeon collected on day 2 post infection (A and B) and brain section collected on day 5 post infection (C and D) are shown. (E and F) Representative immunohistochemical images of the brains infected with Pigeon04 (E) and T.sparrow05 (F). Original magnification: ×10 (A–D); ×20 (E and F).

We compared lesions in the lungs and brains collected from 3 pigeons sacrificed on days 2, 5 and 9 post infection for both HPAIVs. On day 5 post infection, Pigeon04 induced severe encephalitis in 1 of 3 pigeons, whereas T.sparrow05 induced mild encephalitis in 2 of 3 pigeons (Fig. 6C and D). On day 9 post infection, though mild, both viruses induced encephalitis in 1 to 2 pigeons (data not shown). No lesions were observed in the brains infected with either of the two HPAIVs on day 2 post infection (data not shown). Immunohistochemical analysis showed that both viral antigens were detected in cells that were considered to be neuron and glial cells morphologically (Fig. 6E and F). In the lungs, both viruses induced very mild to mild focal non-purulent interstitial pneumonia in 1 or 2 of 3 pigeons on day 2 post infection (Fig. 6A and B). On days 5 and 9 post infection, no lesion was observed with infection of both HPAIVs, except for very mild focal non-purulent interstitial pneumonia in 1 pigeon infected with Pigeon04 on day 5 post infection (data not shown). Viral antigens were not detected in the lungs infected with Pigeon04 and T.sparrow05 throughout the infection periods (data not shown). These data showed that both HPAIVs occasionally induced lesions in the lungs and brains of the infected pigeons, but almost all the lesions were mild.

## Discussion

Proper immune response is essential for host defenses against infection by foreign pathogens. Excessive responses may be detrimental to the host even if elimination of the pathogen is successful. H5N1 HPAIV and 1918 H1N1 viruses have been reported to have increased virulence in mice and macaques accompanied by increased viral replication and aberrant host-cytokine responses, compared to the seasonal influenza A virus. In fact, these viruses markedly induced the expressions of pro-inflammatory cytokines and chemokines including IL6, IL1α, IL1β, TNFα, IFNγ and CCL5 in the lungs, resulting in severe pneumonia [30]–[35]. Although high inductions of Type I interferons (IFNα and IFNβ) were also observed in the lungs of mice and macaques infected with H5N1 HPAIVs, theses viruses robustly replicated in those animals, suggesting that induction of IFNs does not always result in successful elimination of the viruses [32], [35]. In pigeons, the HPAIVs have been reported to establish infection, and could cause respiratory inflammations and encephalitis, even though pigeons are more resistant than chickens against such infections [23], [25]. However, little is known about host immune responses in pigeons against HPAIV infections. Thus, as a first step toward understanding host responses in pigeons against HPAIV infections, we set up quantitative real-time PCR analysis in pigeons and assessed how two Thai HPAIVs induced host immune-related genes in pigeons. We found that Pigeon04 efficiently replicated in the lungs and spread to multiple organs such as brains compared to T.sparrow05, but did not induce robust host immune responses in the lungs of the infected pigeons.

Viral replication and host-cytokine response in the lungs of the infected pigeons appeared different in the respective groups. In the case of T.sparrow05 infection, weak host cytokine responses in the lungs were considered to be merely due to lower viral replication than that of Pigeon04 throughout the collection periods (Table 2 and Fig. 2). In contrast, Pigeon04 induced the mRNA expressions of ISGs such as Mx1, PKR and OAS to at least 10 fold greater extents than T.sparrow05 in the lungs of the infected pigeons on day 2 post infection (Fig. 2). However, their expression levels did not increase in the lungs on day 5 post infection when peak viral replication was observed (Table 2 and Fig. 2). Expressions of pro-inflammatory cytokines (IL6 and IL1β), chemokines (IL8 and CCL5) and IFNα were low regardless of the level of viral replication (Table 2 and Fig. 2). There is a report that high levels of IL6 in bronchoalveolar lavage fluid (BALF) were found in patients who died from acute respiratory distress syndrome compared to those who survived, suggesting that the levels of IL6 are associated with severity of lung inflammation in patients [36]. We previously showed that Pigeon04 highly induced the expressions of pro-inflammatory cytokines such as IL6 and TNFα in the lungs of the infected mice, resulting in severe pneumonia [35]. Thus, it was suggested that the low expression levels of pro-inflammatory cytokines in the lungs following Pigeon04 infection resulted in mild pneumonia (Figs. 2 and 6). It should be noted that the expression of TLR3 was inversely correlated with viral replication in the lungs following Pigeon04 infection (Fig. 4A). One of the pattern recognition receptors, TLR3, detects microbes including the influenza A virus that initiates the innate immune response against foreign pathogen infections [37]. A virulent influenza A virus is reported to induce mRNA expression of TLR-3 in the lungs of infected mice [38]. TLR3-deficient mice infected with the virus had prolonged survival that was accompanied by reduced pro-inflammatory cytokines such as IL6 and RANTES in BALF and reduced lung lesions [38]. Therefore, it was suggested that low levels of TLR3 expression despite efficient viral replication in the lungs may lead to sluggish induction of pro-inflammatory cytokines such as IL6 in the lungs.

In the brains infected with Pigeon04, mRNA expression levels of Mx1, OAS, IL6, IL1β and CCL5 were at least 17 fold higher than those of respective genes in the infected lungs on day 5 post infection when this virus almost similarly replicated in the lungs and brains (Table 2, Figs. 2 and 3). Inductions of these genes upon Pigeon04 infection appeared different in the brains and lungs of the infected pigeons. Differential host responses dependent on organs against infection by pathogen have been reported. Expressions of IFNα and IL12 in the brains of macaques following Simian Immunodeficiency virus infection were down-regulated, whereas they were up-regulated in the lungs of the infected macaques [39]. Koedel et al reported that Myeloid differentiation factor 88 (MyD88), which is known to be the adaptor protein initiating the inflammatory signaling pathways of TLRs, was up-regulated in the brain only of mice infected with *Streptococcus pneumonia* [40], [41]. They also showed that the mRNA expressions of pro-inflammatory cytokines (IL1β and TNFα) and MIP-2 that were observed in the infected brains, but not in the infected lungs of MyD88 knockout mice were dependent on MyD88 [40]. We previously showed ≥250 fold up-regulations of IFNβ, IL6 and MIP-2 in the lungs, but ≤10 fold up-regulations of the same genes in the brains of the infected mice following Pigeon04 [35]. Factor(s) and/or mechanism(s) that determine organ specific responses against the same stimulus need to be explored.

In this study, 25% of the pigeons died within 2 weeks not only after inoculation of the viruses but also after inoculation of the medium, suggesting that laboratory environmental stress may have been the cause of the death. The transfer of wild animals into a captive environment is thought to very stressful for the animals [42]. However, when performing a laboratory experiment on pigeons, it is impossible to handle them without subjecting them to stress. In considering that, we compared host responses of pigeons between mock infected- and virus-infected groups under “the same laboratory conditions”. We found that Pigeon04 replicated in the lungs more efficiently than T.sparrow05, but did not induce excessive expressions of innate immune and inflammatory-related genes in the lungs of the infected pigeons. It was suggested that pigeons could have tolerance towards Pigeon04 infection because of their moderate host cytokine responses following infection. Our study is the first attempt to assess host cytokine responses against HPAIV infection in pigeons, and provides useful information into the relationship between HPAIV pathogenesis and host immune responses in pigeons.

## Materials and Methods

### Virus and animal

Two H5N1 HPAIVs, A/Pigeon/Thailand/VSMU-7-NPT/2004 (Pigeon04) and A/Tree sparrow/Ratchaburi/VSMU-16-RBR/2005 (T.sparrow05), isolated from wild birds through routine surveillance activities during HPAI outbreaks in poultries in Thailand in 2004–2005, were used in this study. Pigeon04 was isolated from a dead pigeon in Nakhon Pathom province in 2004, and T.sparrow05 was isolated from a live tree sparrow in Ratchaburi province in 2005 as mentioned previously [35]. Both viruses belong to clade 1 of the classification system (WHO/OIE/FAO H5N1 Evolution Working Group 2007) in the HA gene, as described previously [35]. Virus stocks were propagated in Madin–Darby canine kidney (MDCK) cells, and the culture supernatant was harvested and stored at −80°C. Viral infectivity of each strain was determined by serial titration of viruses in 10 or 11 day-old embryonated eggs, and was expressed as 50% of the egg infectious dose (EID_50_)/mL by the method of Reed and Muench before use [43]. Adult rock pigeons (*Columbia livia f. domestica*) from a local farm in Thailand that were clinically healthy and serologically negative for H5-specific haemagglutination inhibition (HI) antibodies, were used in this study. All pigeons were housed in isolators (CH8ISOL/CM12ISOL, Allentown Caging Equipment Company, Inc., USA) ventilated under negative pressure with HEPA-filtered air, and acclimated for five days before viral inoculation. The H5N1 HPAIV challenge experiments were performed under the guidelines of the Animal Care and Use Protocol on the approval of The Faculty of Veterinary Science Animal Care and Use Committee (FVS-ACUC) in an animal bio-safety level 3 containment laboratory at Mahidol University, Thailand (Approval No. MUVS-2009-10).

### Experimental design

To examine the survival rate of pigeons infected with the two HPAIVs, groups of eight pigeons were inoculated intranasally with 10^6^ EID_50_ of the HPAIVs or mock infected with medium at a volume of 0.1 mL. After the inoculation, all pigeons were monitored daily for clinical signs or death up to day 14 post infection. Trachea and cloacal swab samples were collected from the dead pigeons and the surviving pigeons on day 14 post infection for virus titration. Blood samples were also collected from the surviving pigeons on day 14 post infection for HI tests. To examine viral distribution, pathology, and host gene response of pigeons infected with the HPAIVs, groups of 9 pigeons were inoculated with the HPAIVs or mock infected with medium as described above. On days 2, 5 and 9 post-inoculation, 3 live pigeons in each group were euthanized to collect tracheal and cloacal swabs in a 2 mL volume of freezing medium. Also, tissues of the brain, lungs, spleen, liver, kidney and rectum were dissected and homogenized to make suspensions of 10% in MEM containing antibiotics.

### Virus titrations in swabs and organs of pigeons infected with the HPAIVs

All swabs and tissue samples were stored at −80°C before titration. The swab samples and tissue samples were titrated in the eggs, and values calculated by the method of Reed and Muench [43] were expressed as EID_50_/mL and EID_50_/g of tissue, respectively.

### Serological analysis

Sera collected from all pigeons before inoculation and virus-infected or mock inoculated pigeons on day 14 post infection were treated with trypsin and potassium periodate to remove any non-specific inhibitor, as described previously [44]. Then, the HI test was performed using the treated sera, goose red blood cells and 4 hemagglutinating units of the H5N1 HPAIVs as the antigens. The pre-inoculation sera of all pigeons used in this study were serologically negative for H5-specific HI antibodies (data not shown).

### Histopathological analysis

The excised tissues of the lungs and brains from the virus-infected or mock inoculated pigeons were fixed in 10% neutral phosphate buffered formalin. Fixed samples were embedded in paraffin, and sectioned. One section was stained with hematoxylin and eosin (HE), and observed microscopically. The other section was immunohistochemically stained with goat anti-influenza A virus polyclonal antibody (OBT1551, AbD serotec, Kidlington, UK) followed by horseradish peroxidase anti-goat Ig conjugate (Histofine Simple Stain, Nichirei Inc., Tokyo, Japan).

### Nucleotide sequencing of immune-related genes in pigeon

Partial sequences of 14 immune-related genes (RIG-I, TLR3, IFNα, Mx1, PKR, OAS, IL6, IL10, TGFβ3, SMAD7, CCL5, Caspase3, ApaF, Bcl2) and β-actin of pigeon were identified in this study to examine host mRNA expressions of pigeons infected with HPAIVs (Table 3). Total RNA prepared from the lungs of uninfected live pigeon or virus-infected dead pigeon was extracted using TRIzol reagent (15596-026, Invitrogen, Carlsbad, CA, USA) according to the manufacturer's instructions, purified in isopropyl alcohol, and diluted in RNase-free water. The RNA was treated with DNase I (1 unit per 1 ug RNA) (Promega, Madison, WI, USA) to remove the residual genomic DNA at 37°C for 1 hr, followed by inactivation of DNase I at 65°C for 10 min. Then, the DNase-treated RNA samples were re-purified using a RNA mini kit. cDNA was synthesized from mRNA with oligo(dT)_20_ primers using the SuperScript™ III First-strand Synthesis System For RT-PCR (18080-051, Invitrogen) according to the manufacturer's instructions. Then, part of each target gene was amplified by PCR with Ex Taq polymerase (RR001B, Takara Bio, Shiga, Japan). Information on the primer pairs to determine partial sequences of 14 immune-related genes is mentioned in Table S1. Primer sequences were chosen based on a conserved region of each gene in birds including chicken (*Gallus gallus*), duck (*Anas platyrhynchos*), goose (*Anser anser*), turkey (*Meleagris gallopavo*), zebra finch (Taeniopygia guttata) or mammals including mice (*Mus musculus*) and human (*Homo sapiens*). The amplified PCR products were purified, and sequenced directly using the Big Dye Terminator sequencing kit, version 3.1 (Applied Biosystems, Foster City, CA, USA) and the primers used above on an ABI Prism 3100 genetic analyzer (Applied Biosystems). The nucleotide sequences determined in this study were submitted to the Genbank database under accession numbers AB618532–AB618546.

### Quantitative real time PCR analysis

Part of the lungs or brains from the virus-infected or mock inoculated pigeons were preserved in RNA later solution (AM7021, Ambion, Austin, TX, USA) and stocked at −80°C before extraction of RNA. Total RNA was extracted from these samples, purified, treated with DNase I and reverse-trancribed to cDNA as described above. The cDNA samples were diluted (1∶10) and used as templates. PCR reactions were performed using equal amounts of cDNA samples with the primers specific for the target genes (RIG-I, TLR3, IFNα, Mx1, PKR, OAS, IFNγ, IL1β, IL6, IL8, IL10, TGFβ3, SMAD7, CCL5, Caspase3, ApaF, and Bcl2), β-actin and SYBR® Premix Ex Taq™ II (RR081A, Perfect Real Time, Takara bio), as illustrated by the manufacturer. The primer pairs of each gene used in the quantitative real-time PCR analysis were mentioned in Table 4. Quantitative real-time PCR analysis was run in triplicate with Cromo4 (Bio-lad laboratories, CA, USA) by the following cycle parameters: 1 cycle at 95°C for 30 sec followed by 40 cycles of 95°C for 5 sec and 60°C for 30 sec. Differences in gene expressions were calculated by the 2^−ΔΔ^Ct method and expressed as fold change in gene expression [45]. β-actin was used as the endogenous control to normalize quantification of the target gene. Average results ± standard deviations were expressed as fold change compared to the uninfected pigeons.

## Supporting Information

Table S1 **Primer sequences to determine partial sequences of 14 immune-related genes.**
(DOC)
