# Supplementary material for: Host Cytokine Responses of Pigeons Infected with Highly Pathogenic Thai Avian Influenza Viruses of Subtype H5N1 Isolated from Wild Birds
Source: PLoS One. 2011 Aug 3;6(8):e23103. doi: 10.1371/journal.pone.0023103 (PMC3149639; doi:10.1371/journal.pone.0023103)
Supplement: Table S1 — Primer sequences to determine partial sequences of 14 immune-related genes. (DOC) [file pone.0023103.s001.doc]

| TableS1. Primer sequences to determine partial sequences of 14 immune-related genes. | |
| --- | --- |
| Gene | Sequence (5'→3') |
| RIG-I |  |
| Forward primer | ACTAAACTCATTGAATG |
| Reverse primer | GATATGGCCTGTATGTC |
| TLR3 |  |
| Forward primer | ATGTTCACAGGTCTGAA |
| Reverse primer | CAAGCAATGCTTTCACAGGT |
| IFNα |  |
| Forward primer | GGAAAAGCCTCCAGCTCCT |
| Reverse primer | CGGACATGGTCCCAGG |
| Mx |  |
| Forward primer | CTGGAACTTAAACTGAA |
| Reverse primer | TCCACCTCTTGAGCCAT |
| PKR |  |
| Forward primer | GCAAARGAAGCATATGA |
| Reverse primer | AATATATTCTGAGGCTT |
| OAS |  |
| Forward primer | CCCAGCTTCACAGAACT |
| Reverse primer | GTCAGCAGCTCCAGTGCATA |
| IL6 |  |
| Forward primer | ACCGTSTGCSAGAACAGCATG |
| Reverse primer | TCARRCACTGAAACTCCTGG |
| CCL5 |  |
| Forward primer | AAGCTGCCYCAGAATCA |
| Reverse primer | TCACTGCAKCTCCAGG |
| IL10 |  |
| Forward primer | CCCWCCTGCCTGCACTTC |
| Reverse primer | TGCCCAGGTCGCCCAT |
| TGFβ3 |  |
| Forward primer | GAAGAACTGCTGCGTGC |
| Reverse primer | TCAGCTGCACTTGCAG |

| Table S1. (continued.) | |
| --- | --- |
| SMAD7 |  |
| Forward primer | GGCTGTACTCTGTCCAA |
| Reverse primer | CAGCAACGTCCTGGAGT |
| Caspase3 |  |
| Forward primer | AGTTACAGAATGGATTATCCAGA |
| Reverse primer | GTTAAAATCTGCATGAGTTC |
| ApaF |  |
| Forward primer | CTTAACATTGAGGAGGC |
| Reverse primer | CAGGCATGGTAAACTGCATC |
| Bcl2 |  |
| Forward primer | AGATAGTGCTGAAGTAC |
| Reverse primer | AGGCATCCCATCCTCCGT |
| β-actin |  |
| Forward primer | TCCGGTATGTGCAAGGC |
| Reverse primer | GTGTTGGTAACAGTCCG |
